# Immediate effect of food intake by the nursing mother on the macronutrient content of colostrum

**DOI:** 10.1016/j.jped.2025.03.004

**Published:** 2025-04-03

**Authors:** Regina C.F.A. Nascimento, Virgínia G.A. Hochman, Camila B.M. da Silva, Bernardo V. do Valle, Yasmin N.di V. do Amaral, Manuela Dolinsky, Alan A. Vieira

**Affiliations:** a Universidade Federal Fluminense, Faculdade de Medicina, Departamento Materno-Infantil, Mestrado Profissional em Saúde Materno-Infantil, Niterói, RJ, Brazil; b FIOCRUZ, Instituto Fernandes Figueira, Instituto Nacional de Saúde da Mulher, da Criança e do Adolescente, Rio de Janeiro, RJ, Brazil; c Universidade Federal Fluminense, Faculdade de Nutrição, Mestrado Profissional em Saúde Materno-Infantil, Niterói, RJ, Brazil

**Keywords:** Human milk, Colostrum, Macronutrients, maternal nutrition, Diet and nutrition

## Abstract

**Objective:**

Human milk has a dynamic composition that is ideal for the needs of infants. However, the factors that affect the nutritional content of human milk are still unclear. This study aimed to evaluate the immediate effect of maternal food intake (lunch) on the macronutrient composition of colostrum.

**Methods:**

This prospective study performed a paired analysis of macronutrient concentrations in the colostrum of healthy postpartum women. Three milliliters of colostrum were collected from 65 participants 30 min before and 2 h after a meal (lunch) by manual expression. The nutritional content of the meal was similar for all mothers. Colostrum analysis was performed using a Human Milk Analyzer (Miris®).

**Results:**

The fat content was significantly higher in colostrum samples collected 2 h after lunch than in those collected 30 mins before lunch (2.3 ± 1.1 vs. 2.8 ± 1.4 *g* %, *p* = 0.002). No significant differences were observed in the protein (1.9 ± 0.7 vs. 1.9 ± 1.0) and carbohydrate (6.4 ± 0.8 vs. 6.4 ± 0.8) content.

**Conclusions:**

Two hours after the mother had lunch, the colostrum fat concentration increased by 20 %.

## Introduction

Breast milk, often regarded as the gold standard in infant nutrition, plays a pivotal role in promoting the health and well-being of newborns. Its unique and multifaceted composition encompasses a plethora of bioactive components, including immunoglobulins, cytokines, growth factors, and enzymes, which collectively confer numerous benefits to the developing infant.^1^ Throughout the course of human evolution, breast milk has undergone intricate adaptations to meet the evolving nutritional requirements of infants, reflecting a harmonious interplay between maternal physiology and offspring needs. Initially, colostrum is produced for a short and limited period, usually within the first 2–5 days after birth, richer in proteins, immunoglobulins, and vitamin A. Over time, it gains new nutritional characteristics, mainly related to an increased amount of total fat, becoming mature milk.^1^^,^^2^

Despite millennia of evolutionary refinements, the precise mechanisms governing the secretion of macronutrients by the mammary gland remain a subject of ongoing investigation. While numerous studies have endeavored to elucidate the composition and functional properties of human milk (HM), significant gaps persist in our understanding of the intricate regulatory pathways that govern milk synthesis and secretion.^3^, ^4^, ^5^ Unraveling the underlying determinants of macronutrient secretion holds profound implications for optimizing infant nutrition and fostering optimal growth and development.

One prominent determinant of HM composition is maternal dietary intake, which exerts a direct influence on the nutritional profile of breast milk. Maternal diet serves as the primary source of nutrients for lactating women, thereby impacting the quantity and quality of nutrients available for being incorporated into breast milk. However, the relationship between maternal dietary patterns and HM composition is complex and multifaceted, influenced by a myriad of factors including maternal metabolic status, nutrient bioavailability, and dietary diversity.^1^^,^^6^, ^7^, ^8^, ^9^, ^10^, ^11^, ^12^

Despite the wealth of research exploring the impact of maternal diet on HM composition, methodological inconsistencies across studies pose significant challenges to the interpretation and generalization of findings. Variations in study design, dietary assessment methods, and sample collection protocols complicate efforts to establish clear associations between maternal dietary patterns and HM composition. Furthermore, the dynamic nature of HM composition, which undergoes temporal fluctuations influenced by factors such as lactation stage and diurnal variation, further complicates efforts to delineate the precise effects of maternal diet on HM composition.^13^, ^14^, ^15^, ^16^, ^17^ Given the potential implications for infant health and development, there is a pressing need for rigorous and well-controlled studies examining the immediate effects of maternal dietary intake on HM composition, particularly during the critical postpartum period. By elucidating the acute effects of maternal diet on the macronutrient content of human colostrum, the present study aims to contribute to our understanding of the dynamic interplay between maternal nutrition and infant feeding outcomes. Through a comprehensive evaluation of the immediate effects of maternal food intake on HM composition, the authors seek to inform evidence-based strategies for optimizing maternal nutrition and promoting optimal infant health outcomes.

## Methods

This was a before and after study, wherein the concentrations of macronutrients in the colostrum of healthy postpartum women were compared before and after lunch. All postpartum women hospitalized in two public maternity hospitals between January 2017 and October 2019 who delivered term babies in the 5 days before sample collection were included in the study. Puerperal women who had gestational or pre-gestational pathology and those for whom breastfeeding was contraindicated, such as mothers with human immunodeficiency virus and human T-lymphotropic virus infection, were excluded. The recruitment of research participants was carried out at least 12 h after birth and their inclusion in the study followed the sequential order of hospitalization until reaching the n-sample that was calculated. There were no dropouts. The vast majority of samples were collected between 36 and 48 h after birth. Samples collected after this period only included women who needed to extend their hospital stay (2 samples).

During hospitalization, all postpartum women treated in the hospitals studied have their food offered by the institution's nutrition service in a restricted manner, following the institution's dietary protocols. Lunch was composed of 35 g of protein, 85 g of carbohydrates, and 20 g of lipids, with a total caloric value of 650 kcal. Water intake was allowed on demand. The hospital diet is standardized and does not allow external foods, with the aim of ensuring the safety, efficacy, and recovery of the patient, preventing dietary risks, and ensuring the effectiveness of medical treatment.

Colostrum samples (3 ml) were obtained from the same postpartum woman twice on the same day; the first sample was obtained 30 min before lunch (11:30am), and the second was obtained 2 h after lunch (02:00 pm). The choice of which breast (right or left) would be milked was made by simple randomization; the second milking was performed on the same breast chosen previously. Manual milking was performed by the puerperal woman herself or by the researcher without any breast preparation; an interval of at least 40 min was allowed after the last breastfeeding. The collected samples were stored in glass vials and immediately sent to the milk bank for freezing at −20 °C.

Colostrum analysis was performed using a Miris® Human Milk Analyzer (Miris, Sweden) after thawing, heating, and homogenization of the samples. In addition to the carbohydrate, fat, and protein content of the samples, the amount of calories was also calculated. The device was cleaned and calibrated after every 10 readings, according to the manufacturer's instructions.

Maternal sociodemographic, pre-gestational, and current anthropometric data (weight, height, and body mass index) were collected. The information on the newborns, such as weight, gestational age at birth, adequacy of weight to gestational age, and Apgar score was obtained from the medical records.

The sample size was based on variations in the lipid content, the macronutrient that presents the greatest variability in HM. The authors considered a difference between groups of 0.5 *g* % in lipid concentration, a standard deviation of 1.2 *g* %, α error of 5 %, and β error of 20 %. Therefore, 130 samples of colostrum (65 samples before lunch and 65 samples after lunch) donated by 65 participants were included.

Categorical variables are presented as absolute and relative numbers. Continuous variables are presented using measures of central tendency and were compared using paired *t*-tests. The statistical package IBM® SPSS® version 16.0 was used, and a p-value < 0.05 was considered significant.

## Results

The sociodemographic and obstetric characteristics of the 65 mothers and newborns who participated in this study are summarized in Table 1.

**Table 1:** Sociodemographic and obstetric characteristics of mothers and newborns were included in the research.

	Mean ± SD *N* = 65	Range
Maternal age (years)	23 ± 4	16–31
Maternal education (years)	10 ± 3	3–15
Income (dollars/year)	4.164 ± 2.950	960–13.850
Parity	2 ± 1	1–7
Number of prenatal appointments	8 ± 3	2–17
Weight gain during pregnancy (kg)	11.6 ± 5.0	1.6–20.6
Body mass index (kg/m^2^)	30 ± 5	20–46
Infant birth weight (g)	3.250 ± 580	2.130–4.470
Gestational age (weeks)	39 ± 1	37–41

SD, standard deviation.

The average maternal age was 23 ± 4 years and the duration of maternal education up to the time of the study was 10 ± 3 years; annual income was 4164 ± 2950 dollars. The average parity was 2 ± 1 and the number of prenatal appointments was 8 ± 3. Weight gain during pregnancy was 11.6 ± 5.0 kg and body mass index at birth was 30 ± 5 kg/m2. Regarding newborns, the baby's birth weight was 3250 ± 580 g and the gestational age at birth was 39 ± 1 week.

As this was a paired study, in which each nursing mother was under her own control, the clinical and sociodemographic characteristics of the mothers and their newborns did not interfere with the results.

The carbohydrate (6,4 ± 0,8 vs 6,4 ± 0,8 g/dL) and protein (1,9 ± 0,7 vs 1,9 ± 1,0 g/dL) colostrum concentrations did not differ before and after lunch. However, the concentrations of fat (2,3 ± 1,1 vs 2,8 vs 1,4 g/dL) and energy (57,1 ± 11,6 vs 31,7 ± 15,6 kcal/dL) were 22 % and 8 % higher, respectively, in the samples collected 2 h after maternal food intake than in the samples collected 30 min before lunch (Table 2).

**Table 2:** Comparison of macronutrient concentration of colostrum in the pre- and post- prandial periods.

	Pre-prandial period n:65		Post-prandial period n:65		
Macronutrients	Mean ± SD	Median (IQR)	Mean ± SD	Median (IQR)	*p* value
Carbohydrates (g/dL)	6.4 ± 0.8	6.5 (6.2–6.9)	6.4 ± 0.8	6.5 (6.3–6.8)	0.930
Protein (g/dL)	1.9 ± 0.7	1.6 (1.5–1.9)	1.9 ± 1.0	1.6 (1.4–1.8)	0.767
Lipids (g/dL)	2.3 ± 1.1	2.1 (1.4–3.0)	2.8 ± 1.4	2.6 (1.6–3.2)	0.002
Calories (kcal/dL)	57.1 ± 11.6	55 (49–63)	61.7 ± 15.6	58 (53–66)	0.010

SD, standard deviation; IQR, interquartile range.

## Discussion

In this study, an immediate effect of maternal food intake on colostrum composition was observed 2 h after lunch: the fat concentration increased by 20 %.

Colostrum is usually produced within the first 2–5 days after birth. This time is correlated to the postpartum woman's stay in the hospital, and this allowed controlling some variables described as possible factors of interference in the dynamics of the production and secretion of macronutrients by the breast acini, such as the moment and way of collection, and mainly the nutritional intake. The window of time in which colostrum can be collected and studied is narrow, making it a priority research topic to better understand how it contributes to neonatal health and child development.

The timing of colostrum collection, specifically 2 h after maternal dietary intake, was based on established physiological processes governing lipid digestion and absorption kinetics. Lipid metabolism involves a sequence of events, starting with emulsification in the stomach, followed by enzymatic hydrolysis in the small intestine, and ultimately absorption into the bloodstream via the intestinal mucosa. Studies have demonstrated that the postprandial period, particularly the initial hours following lipid ingestion, is characterized by heightened lipidemia and chylomicron formation, indicative of active lipid digestion and absorption. Notably, the concentration of lipids in breast milk has been shown to correlate with maternal serum lipid levels, peaking at 2–4 h after a meal.^18^, ^19^, ^20^

Furthermore, the composition of breast milk, including lipid content, exhibits diurnal variations influenced by maternal dietary patterns and metabolic rhythms.^13^^,^^21^, ^22^, ^23^ By sampling colostrum 2 h postprandially, the authors aimed to capture the acute effects of maternal dietary intake on lipid secretion into breast milk, thereby elucidating the immediate impact on neonatal nutrition and development. Overall, the chosen time point aligns with the physiological kinetics of lipid digestion and absorption, optimizing the sensitivity of the methodology to alterations in colostrum composition in response to maternal dietary manipulation.

Concerning the immediate influence of maternal nutrition on the concentration of macronutrients in HM, Ward et al. observed that an increase in sugar and fat intake led to a sharp increase in the composition of these macronutrients, mainly fat, in breast milk. In addition, these authors also noticed a variation in the concentration of proteins in HM regardless of food intake, suggesting a “circadian cycle” of concentrations of this macronutrient, which has been described previously.^24^^,^^25^

The relationship between nutrient intake and its presence in HM is difficult to prove since several factors influence the physiology of milk production. Among these, some are already known and widely studied.^26^ However, the fat concentration of HM is directly related to maternal body fat reserves, local synthesis by the mammary gland from fats in the plasma, and maternal food intake.^27^

Maternal intake and the nutrient composition of HM are closely but complexly related. Factors such as maternal diet quality, maternal metabolic status, nutrient availability, and dietary diversity are known to influence milk composition. However, the exact relationship between these factors and the nutrients present in HM is still being explored.

In this particular study, colostrum collected 2 h after lunch revealed a 20 % increase in fat concentration, without significantly altering protein and carbohydrate levels. This suggests that the presence of fat in milk may be directly related to maternal lipid absorption and the release of these lipids into the bloodstream, which in turn may be transferred to colostrum.^28^

The adaptation of human milk over time, with increasing fat and changing composition, reflects an evolutionary response to the nutritional needs of the infant as it grows and develops. In colostrum, this fat transfer is a fundamental part of the infant's initial adaptation to extrauterine life, providing a concentrated source of energy.^28^^,^^29^

Among the methodological challenges, difficulties in verifying the participants' food intake prior to the study are noteworthy. Often, controlled food intake is ensured by collecting recall information from the participants, which is not always reliable. In the current study, the food intake of the research participants was controlled because the hospital prohibited the entry of foods other than those provided by the hospital staff.

Other difficulties are related to the method of collection of HM samples (time of day, relationship with breastfeeding, collection method–by manual expression or by an electric pump, and storage method) and factors related to breastfeeding, such as the presence of diseases, age, and use of medication.^30^, ^31^, ^32^

In the present study, several of these variables were controlled. All pregnant women were healthy, did not use any medication, were of a similar age, and had a full-term pregnancy without intercurrences. Further, samples were collected at the same time of day using the same technique, before and after eating a meal with similar nutritional content. In addition, samples from the same nursing mother were analyzed in pairs, that is, the nursing mother was her own control.

These methodological considerations presented in this study are essential for the robustness and reliability of the results. The study was designed to minimize other factors that could affect colostrum composition. The choice to collect colostrum samples before and after maternal feeding and the strict control over the nutritional content of the meal ensured that the observed differences in colostrum were attributed to the direct effect of feeding and not to variations in diet.

Another important methodological point was the fact that each participant served as her own control, which helped to eliminate the influences of individual factors such as weight, age, or health status. Furthermore, control over the consumed meal, the exact time of collection and the exclusion of participants with pathologies that could interfere with milk production are measures that ensure the homogeneity of the sample and reduce the possibility of bias.

Most studies on the correlations between maternal diet and concentration of nutrients in HM have shown conflicting results, often explained by the difference in methodologies.^14^^,^^33^, ^34^, ^35^, ^36^ However, an interventional study published in 1998 aimed to investigate the immediate effects of a single dose of six different types of fats on the quality and concentration of fatty acids in HM. In this study, the influence of the administration of fatty acids was perceived in the first analysis of HM 6 h after the ingestion of fats. The peak concentration of fatty acids in milk was observed between 10 and 24 h after ingestion and remained significantly elevated for up to 3 days. Similar findings were reported by Insull and Hirsch^37^ in 1958.

The influence of some factors on the variations in the concentrations of macronutrients in HM is already clear. Moran-Lev et al.^22^ and Lubetzky et al.^13^ reported the circadian influence on fat and energy concentrations of HM during the first 7 weeks of lactation in mothers of preterm infants. Milk samples were collected in the morning and at night, and the fat and energy concentrations were significantly higher at night. In both studies, lactose and protein concentrations did not change significantly.

The influence of circadian rhythms on the fat concentration of HM is still not fully understood. A high frequency of breastfeeding or increased food intake by the mother during the day, preceded by night fasting, could explain this variation.^13^^,^^22^^,^^23^

An uncontrolled element in this study was the number of feedings offered to infants during the study period. The mothers continued to breastfeed on demand, as it would have been unethical to deprive the newborns of this benefit. Of note, the colostrum samples were analyzed after controlled maternal food intake. Therefore, the present results cannot be extrapolated to breastfeeding mothers with uncontrolled food intake or to samples of mature milk.

Nutrient metabolism during lactation is closely linked to the process of milk secretion. Lipogenesis is activated after lipid ingestion, increasing the amount of fat transported into breast milk.^38^ In addition, the immediate effect of maternal feeding on colostrum, specifically on fat content, can also be analyzed from the perspective of nutrient bioavailability. Bioavailability is the degree to which nutrients consumed by the body are available for absorption and utilization. Maternal nutrition directly affects the amount of nutrients that are metabolized and, consequently, incorporated into milk, and the lipid composition of milk, as demonstrated by the increase in colostrum fat concentrations after a meal, reflects this efficient absorption and utilization of lipids.^28^^,^^39^

The relationship between food intake and the protein and carbohydrate composition of milk was not observed in this study, since there were no significant differences in protein and carbohydrate levels before and after the meal. This finding may be explained by the complexity of the regulation of protein synthesis in breast milk, which appears to be more stable and less influenced by immediate food intake than lipids. Protein production in milk may be more dependent on chronic factors, such as the mother's general nutritional status, than on the punctual intake of nutrients.^38^

These studies reinforce the importance of the quality of maternal nutrition in the composition of macronutrients in breast milk, especially fat. The observed increase in colostrum fat 2 h after meal suggests that collecting breast milk at specific times may be a useful strategy to increase the caloric value of milk, especially in human milk banking programs. In addition, understanding the mechanisms governing the secretion of macronutrients into breast milk has implications for nutritional counseling for lactating women. By informing mothers about the impact of their diet on milk composition, it is possible to optimize the nutritional quality of breast milk according to the specific needs of the infant.

Understanding how nutrients in the maternal diet influence macronutrient secretion in HM is essential for improving infant nutrition strategies and supporting the healthy development of newborns.

Despite some limitations, the present study showed an immediate effect of maternal food intake on colostrum composition, specifically its fat content, which offers valuable insights into the dynamic nature of human milk (HM) and how maternal diet can influence its composition.

## Ethical approval and consent to participate

The study was approved by the Committee of Ethics in Research with Human Beings of the Medicine School of the Federal Fluminense University (Consubstantiated Opinion: 2.172.260; certificate of Submission for Ethical Appreciation number: 67111517.0.0000.5243) and informed consent was obtained from all participants.

## Data statement

The data is stored on the contact actor's personal computer and can be accessed if necessary.

## Funding source

None.

## Conflicts of interest

The authors declare no conflicts of interest.
